# Cairo Consensus Statement on Research Integrity of Randomised Clinical Trials

**DOI:** 10.12669/pjms.40.8.9779

**Published:** 2024-09

**Authors:** Patrick FW Chien, Khalid S Khan, Mohamed Fawzy, Yacoub Khalaf, on behalf of the Cairo Consensus Group on Research Integrity

**Affiliations:** 1 Patrick FW Chien, Department of Obstetrics & Gynaecology, RCSI & UCD Malaysia Campus, 10450 Penang, Malaysia; 2 Khalid S Khan, Department of Preventive Medicine and Public Health, University of Granada, Faculty of Medicine, Granada, Spain. CIBER Epidemiology and Public Health, Madrid, Spain; 3 Mohamed Fawzy, IbnSina (Sohag), Banon (Assiut), Qena (Qena), Amshag (Sohag) IVF Facilities, Egypt; 4 Yacoub Khalaf, Department of Obstetrics & Gynaecology, Guy’s & St Thomas’ Hospital Foundation Trust, London, United Kingdom

Published randomised clinical trials, their systematic reviews and the resulting guidelines compare beneficial and non-beneficial outcomes of interventions to underpin evidence-based patient care.^1^ Research integrity is a multifaceted concept including adherence to ethical and professional principles and standards applied at the various stages of the trial lifecycle from conception and design to conduct and reporting of the results.^2^ Ethics committees assess and approve trial proposals. Research governance offices oversee compliance with standards during the course of the trial. Journal editors and peer-reviewers assess integrity as part of their evaluation before trial publication and remain open post-publication to being alerted about integrity concerns by readers.^3^ In evidence-based medicine, critical appraisal and study quality assessment tools have been developed with validity and reliability, not integrity, in mind. Trial integrity has been under scrutiny as trial retractions and expressions of concern have recently come under the spotlight, undermining trust in trials.^4^-^6^

There is a growing recognition that trials without integrity are slipping through the traditional peer review process.^7^ For this reason, a multi-stakeholder international expert consensus on the integrity of randomised clinical trials, the first of its kind, was conducted to provide best practice recommendations for the various stakeholders involved in the design, conduct, analysis and reporting of such studies.^8^ This process involved synthesising published evidence from 55 systematic reviews on research integrity applicable to clinical trials^9^ followed by two rounds of modified Delphi electronic survey and a final face-to face meeting with 30 stakeholders representing 15 countries from 5 continents (including trialists, ethicists, methodologists, statisticians, consumer representative, industry representative, systematic reviewers, funding body panel members, regulatory experts, authors, journal editors and peer-reviewers) to arrive at the final statement set.

This integrity statement is underpinned by evidence of the published literature as well as being endorsed by a wide range of stakeholders including trial experts, guideline developers and patient representatives. Here, we have reprinted this statement (Table-I) to draw attention to the need for the future global trial research integrity landscape to be protected through improved practice. Funders, institutions (universities and hospitals), professional societies, journals and other stakeholder organisations can use this statement to create discipline-specific guidance to embed integrity culture for clinical research taking place under their umbrella.

**Table-I:** Cairo consensus statement on randomised clinical trial integrity.^8^

*General*
1.Clinical trial integrity guidelines and policies must be explicit, visible, and prospectively enforceable at all levels through an implementation plan.
2.Trialists, ethics committee members, journals editors and peer-reviewers should receive appropriate methodological and integrity training.
3.Trial ethics committees should have accreditation and regional, national and international harmonisation of ethics assessment criteria and review process.
4.There should be continuous public documentation of trials during the entire study lifecycle.
5.Journals should support adoption of responsible research practices in the design, conduct, analysis, reporting and archiving of trials.
6.Institutions should avoid excessive publication pressure.
***Design and approval***
7.Ethics approval should be obtained for all trials, including those using de-identified data.
8.Informed consent should be developed with patient (or their representative) and public involvement.
9.Informed consent should be examined and approved by the ethics committee.
10.Informed consent should include explicitly how the de-identified data will be shared at the time of publication or used for future analysis
11.Trials should be prioritised and resourced according to local health care needs, strategy, and culture, especially in multi-country trials including low-resource settings.
12.Trials should be approved according to local ethics and regulatory framework, especially in multi-country trials including low-resource settings.
13.Translations of patient reported outcomes should be culturally sensitive in multi-country trials including low-resource settings.
14.Equality, diversity and inclusion should be embedded in trial design to maximize generalisability of findings.
15.Sample size estimation should be sufficiently detailed to permit replication.
16.Primary and secondary outcomes should follow the internationally agreed core outcomes whenever available.
17.The trial protocol, including ethics approval, should be prospectively registered with an open-access trial registry prior to participant recruitment. This policy should be included in research institutions` and sponsors` regulations, and researcher employment and funding contracts.
***Conduct and monitoring***
18.Trial site assessment should put in place measures to mitigate integrity breaches with the support of local research governance departments.
19.There should be promotion of admission of honest or unintentional errors in the conduct of the trial without fear of blame. A part of this policy should be training.
20.Innovative recruitment strategies should be participant-driven and should comply with ethics principles.
21.Routinely collected data should be validated before analysis and reporting.
22.Informed consent oversight should be part of trial audit.
23.The membership of independent trial steering and data monitoring committees should declare any potential conflict of interests.
24.The membership of independent trial steering committees should include patient and public stakeholders.
25.Minutes of the independent trial steering and data monitoring committees should be available when required.
26.Data monitoring committee charter should include responsibility for data integrity.
27.Centralized monitoring and selective source data verification should be deployed for ensuring data integrity.
28.There should be transparency in the method(s) of handling missing data at all stages of monitoring and reporting.
29.Early termination of a trial should be undertaken with the input of the independent trial steering and data monitoring committees.
30.Any amendment to study protocol should be reported to the trial registry (with dates). Major changes also require ethics approval.
31.The statistical analysis plan should be developed and published at the start or during the early stages of the trial before the data is made available to the investigators.
32.All analyses should be pre-specified from the outset (the analysis of the primary outcome and secondary outcomes, sub-group analyses, and sensitivity analyses).
33.There should be a single primary outcome pre-specified; when there are multiple key outcomes, valid testing strategies should be considered for maintaining familywise type-1 error within the acceptable limit of 5 %.
34.Trial funders should mandate in their contract with researchers that outcomes are analysed and reported according to preregistration.
35.Databases for trials should include auditable access logs and permission management systems to prevent illicit access to data or editing of data.
36.Trial integrity and quality evidence synthesis both require the avoidance or minimisation of bias in trial conduct.
***Reporting of protocols and findings***
37.Trialists are strongly encouraged not to submit to a predatory journal, avoiding journals without transparency and integrity.
38.Journals’ authors’ instructions should explicitly and comprehensively cover the requirements for openness and transparency.
39.Journals´ electronic submission system should facilitate compliance with the integrity-related authors` instructions.
40.Professional medical writing could help in reporting more clearly and succinctly to meet the integrity requirements. Its contribution should be reported.
41.The speed with which editorial and peer-review decisions are made should be balanced against the possibility of future complaints and retraction.
42.Reporting of ethics approval and informed consent details should be obligatory part of reporting guidelines and authors’ instructions.
43.Ethics or independent data monitoring committee should provide confirmation that the trial was conducted as planned.
44.Authorship contribution (credit according to international guidelines) should be made explicit in the manuscript.
45.Trial protocol and statistical analysis plan should be submitted in unredacted form along with data set, statistical syntax and analytical outputs.
46.Reporting of conflict of interests, funding sources and payments received by all authors should be standardised.
47.Declaration of conflict of interest, funding sources and payments should be mandatory for peer-reviewers and editors.
48.Reporting of patient and public involvement in the trial should be mandatory.
49.Manuscripts should be prepared according to standard reporting guidelines (e.g SPIRIT, CONSORT, GRIPP-2, etc) and their specific extensions for particular trial types (e.g. human challenge trials, trials of social and psychological interventions, etc.).
50.Plagiarism checks should be routinely carried out on the article main text.
51.Errors, deviations from protocol, losses to follow-up, missing outcome data and solutions applied should be transparently reported.
52.Reporting the use of data monitoring committees, its responsibilities and its membership should be mandatory.
53.Among trials conducted in various languages use of translations in patient reported outcomes should be explicit.
54.Primary and secondary outcomes should be mandatorily linked to prospectively registered outcomes.
55.Spin in writing to misrepresent, overinflate or distort the methods, findings, results and conclusions should be eliminated.
56.The strengths and limitations of the integrity-related issues, as well as any flaws in terms of less-than-ideal method implementation that was unavoidable, should be discussed in the manuscript.
***Post-publication***
57.When a post-publication review detects integrity breaches, the implication is that the scientific process failed, so the focus should be on correction and learning lessons openly and collectively.
58.Journals have the responsibility to conduct their pre-publication assessments and peer-review in a manner so as to minimise the risk of post-publication dishonesty allegations.
59.Any guidance concerning post-publication integrity concerns (e.g, COPE https://publicationethics.org, https://doi.org/10.24318/o1VgCAih, https://doi.org/10.24318/cope.2019.2.4) should explicitly emphasise the investigators` responsibility to evaluate the integrity of the complaint and to support the trialists.
60.Institutions and journals should be equally supportive to the complainant(s) and author(s) in handling such complaints. There is a responsibility to protect honest trialists against harassment.
61.Trialists must engage with any request for an explanation for apparent data discrepancy if required by the journal during both peer-review and post publication stages, or by systematic reviewers during evidence synthesis.
62.Trialists have the responsibility to keep detailed records of their trial including original protocol (with any subsequent amendments), ethics approval, details of the trial registration, de-identified raw data set, randomisation sequence employed, statistical plan, syntax and outputs of all the statistical analyses in case these are required to address any post-publication complaints.
63.Declaration of conflicts of interest, funding sources and payments should be mandatory for complainants.
64.Journals should act in an unbiased fashion transparently managing the conflict of interest of their own editors and advisors handling complaints.
65.Trialists, with their institutional input, should be permitted to provide independent expert reports to the journal investigating a complaint.
66.If honest mistakes are identified in post-publication, an erratum should be published.
67.Retraction notices should be clear and interpretable.
68.Post-retraction management of trials with proven misconduct should be based on a system that avoids continued citation and data misuse.
***Future research and development***
69.Educational effectiveness of integrity training should be evaluated.
70.The factors influencing participant willingness to give consent for data sharing should be evaluated.
71.The minimum requirement for adequate informed consent should be established.
72.The criteria for and level of data auditing required during conduct of trial should be delineated.
73.The integrity remit of data monitoring committees should be clarified.
74.The best method(s) for publication credit (authorship contribution) should be determined.
75.Effective peer review models should be developed for evaluation of trials.
76.Automated checks for compliance with reporting guidelines items (e.g CONSORT, SPIRIT, GRIPP-2) should be developed.
77.For the raw data to be shared, journals should clarify the requirements, e.g. randomisation sequence, cleaned or original de-identified dataset, statistical codes, etc.
78.The validity of early post-submission and post-publication integrity tests should be evaluated.
79.A common research terminology should be developed for prevention of selective reporting.
80.Evidence syntheses of trials using reported study-level (not raw) data should develop methods (e.g. subgroup meta-analyses or meta-regression) to evaluate integrity concerns.
81.Evidence syntheses of trials should develop methods to access patient-level (raw) data to maximize transparency.

***Notes:*** Reproduced by permission from Wiley.

The statement presented here is the first of the Cairo consensus series. The initial review for this consensus was conducted in the late 2021, culminating in a final round being held in Cairo, Egypt, in February 2022. This inaugral consensus work addressed research integrity issues relating to the different stages of the entire randomised clinical trial lifecycle. These statements are also currently being translated into Spanish, Arabic and French in order to aid further dissemination. Subsequent to this, two further consensus projects have also been conducted using a similar methodology with the specific focus on post-publication and ethical issues in 2023^10^,^11^ and 2024^12^ respectively. The composition of the consensus group for each project differed to reflect the topic specific expertise required for the issues to be addressed for each particular year. The output from these later projects are either currently undergoing peer review for publication^10^,^11^ or being written up for submission to be published.

The statements from this work are not set in stone and subject to be amended in light of new evidence and developments. Generating this statement is only an initial step as a collective effort on the part of researchers, editors, peer reviewers and other individual stakeholders brought together in the Cairo consensus. Going forward, institutions, human research ethics committees, research governance departments, funders as well as journals will need to endorse and implement the statement in order to achieve the aim of more openness and transparency in clinical trials as part of the broader science integrity agenda.^13^

## Authors Contribution:

**PFWC** wrote the initial draft, edited and approved the final manuscript.

**KSK**, **MF** and **YK** edited and approved the final manuscript.
